# Two modes of exocytosis in an artificial cell

**DOI:** 10.1038/srep03847

**Published:** 2014-01-24

**Authors:** Lisa J. Mellander, Michael E. Kurczy, Neda Najafinobar, Johan Dunevall, Andrew G. Ewing, Ann-Sofie Cans

**Affiliations:** 1 University of Gothenburg, Department of Chemistry and Molecular Biology, 412 96 Gothenburg, Sweden; 2 Chalmers University of Technology, Department of Chemical and Biological Engineering, 412 96 Gothenburg, Sweden

## Abstract

The details of exocytosis, the vital cell process of neuronal communication, are still under debate with two generally accepted scenarios. The first mode of release involves secretory vesicles distending into the cell membrane to release the complete vesicle contents. The second involves partial release of the vesicle content through an intermittent fusion pore, or an opened or partially distended fusion pore. Here we show that both full and partial release can be mimicked with a single large-scale cell model for exocytosis composed of material from blebbing cell plasma membrane. The apparent switching mechanism for determining the mode of release is demonstrated to be related to membrane tension that can be differentially induced during artificial exocytosis. These results suggest that the partial distension mode might correspond to an extended kiss-and-run mechanism of release from secretory cells, which has been proposed as a major pathway of exocytosis in neurons and neuroendocrine cells.

The primary route of neuronal communication involves the extracellular release of small neurotransmitter molecules that are packaged in secretory vesicles. Chemical release is accomplished via fusion of these vesicles with the cell membrane in a process known as exocytosis. A model of exocytosis in membrane blebs from live cells is presented here demonstrating release via two distinct exocytosis mechanisms resulting in either full or partial release.

It was initially assumed that the vesicle completely collapses into the cell membrane following fusion and thereby releases its entire content. However, there is evidence that vesicles often fuse in a kiss-and-run mechanism, where release occurs though a pore followed by its re-sealing^1^,^2^,^3^,^4^,^5^,^6^. Recent results from our group and others suggest that transient fusion with an extended release of part of the vesicle content is in fact the predominant form of release in model cell types^7^,^8^,^9^,^10^. Omiatek et al developed a method termed electrochemical cytometry to measure the neurotransmitter content of single vesicles^11^. When the method was applied to PC12 cells, the amount contained in the vesicles was found to be significantly larger than the amount released per single exocytosis event as measured with amperometry^9^. In fact, an average of 40% of the vesicle content appears to be released in a typical exocytosis event in these cells. The method has also been used to analyze the dopamine content of rat striatal vesicles with the finding that the average vesicle contains significantly more neurotransmitter than previously estimated based on amount expelled, indicating fractional release from these cells as well^10^. Further supporting the idea of partial release, the amount of transmitter released has been shown to be altered rapidly by external factors that are not likely to affect loading and recruitment of vesicles^12^,^13^,^14^,^15^. We have recently presented evidence for exocytosis to occur through an extended kiss-and-run mechanism where the initial nanometer sized fusion pore is believed to expand transiently to then reform into a nanotube following release^8^. It is likely that exocytosis in cells occurs through several different release modes; however, it would be of great interest to determine which mode is the dominant one and what factors dictate and regulate the mode of release that is employed.

We have previously established a minimalistic artificial cell model to study the membrane dynamics of the exocytotic process^16^,^17^. These model systems are large compared to the structures involved in exocytosis, however the effects of membrane properties of this artificial system should scale down to the smaller real system. One important property that might not translate well from the living system is the effect of spontaneous curvature as the model system contains much less curvature by virtue of the larger size. The method was developed using soybean lipid giant unilamellar vesicles (GUVs). In this model, a micropipette is electroporated through both walls of the vesicle. The pipette is then retracted back into the vesicle, bringing with it a lipid nanotube. When pressure is applied though the micropipette, a daughter liposome is formed from the nanotube. This assembly, consisting of a daughter liposome, a nanotube, and a GUV simulates the secretory vesicle, the fusion pore, and the plasma membrane structure formed during exocytosis. The daughter liposome is microinjected with a solution containing the electroactive neurotransmitter molecule dopamine. The vesicle is inflated until the full distention of the nanotube into the giant liposome occurs resulting in the complete release of the vesicle content, mimicking the final stages of exocytosis. Chemical release in these events can be studied using amperometry at a carbon fiber electrode placed against the giant liposome at the position of release. This results in a peak similar to recordings at cells undergoing exocytosis for attomoles of artificial transmitter released. Much knowledge of the exocytotic process can be gained through the exploration of artificial cell models; however, present models of vesicle fusion and exocytotic release mimic the full distension mode with complete release of the vesicle content.

In this paper we investigate release of neurotransmitter using an artificial cell constructed from PC12 cell plasma membrane vesicles, also referred to as blebs, to observe two distinct modes of release, full and partial distension of the fusion pore. By applying our method to PC12 cell blebs we are able to take our model one step closer to the conditions in the living cell giving us further insights into the vital process of neurotransmitter release. The partial distension mode demonstrates the ease with which the membrane forms these structures that mimic the recently presented, but still controversial, mode of extended kiss-and-run where the fusion pore is hypothesized to only transiently expand^8^. Furthermore, these observations provide us with an advanced artificial system for studying both full and partial release of the vesicle content, possibly corresponding to the full and kiss-and-run exocytosis that is believed to occur in most secretory cells.

## Results

### Observation of two release modes during artificial exocytosis

We employ an artificial cell model previously developed using pure soy lipid preparations to study expansion of the fusion pore formed during exocytosis and the subsequent release of neurotransmitter^16^. In this paper we use plasma membrane vesicles, also commonly referred to as blebs, from PC12 cells to construct our artificial cell and vesicle membrane. Blebs have been shown to possess a lipid composition representative of the plasma membrane, however, it is not clear whether the membrane keeps its asymmetric distribution of phospholipids with respect to the two bilayers^18^. The method used here for bleb formation, a combination of formaldehyde and dithiothreitol (DTT), has previously been shown to minimize the formation of protein aggregates on the bleb surface^19^. While the functionality of membrane proteins is not relevant to our experiments, as we are studying a minimalistic model of exocytosis that is independent of protein function, this fact is still important, since aggregation of surface components would most likely result in an altered membrane behavior.

**Figure 1:**
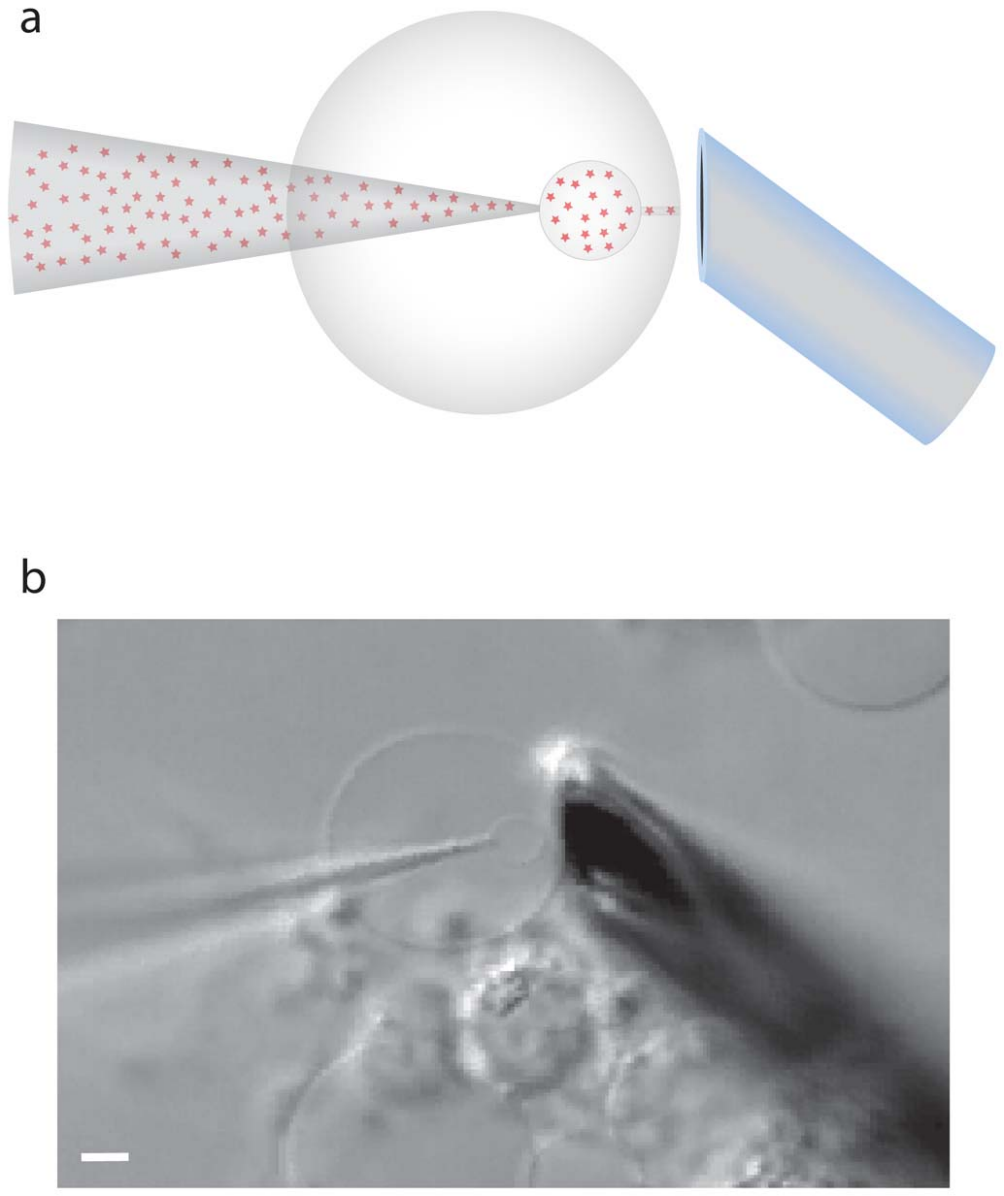
Experimental protocol. (a). Schematic of the experimental setup. The red stars symbolize the dopamine molecules. (b). Picture of the experimental setup with a micropipette inflating a daughter vesicle from the PC12 cell plasma membrane vesicle. Scale bar, 2 μm.

**Figure 2:**
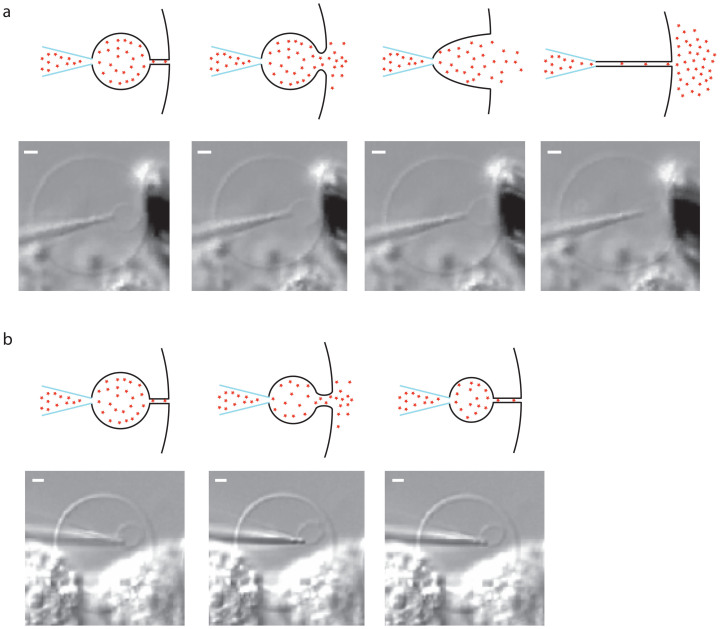
Schematic and micrographs of the two release modes. (a). The full distension mode, where the vesicle opens up to a frustum, which then collapses into a nanotube resulting in the complete release of the vesicle content. Scale bars, 1 μm. (b). The partial distension mode where the nanotube opens up to a larger pore followed by its re-closing to a nanotube. The partial distension results in incomplete release of the vesicle content. Scale bars, 1 μm.

A schematic of the experimental design and a picture of a plasma membrane vesicle under manipulation are shown in Figure 1. The small size of the plasma membrane vesicle inherently makes the daughter vesicles smaller than the vesicles that are formed when using GUVs. The plasma membrane vesicles have an average diameter of approximately 10 μm, whereas the daughter vesicles formed range from 1 to 4 μm in diameter. In comparison to chemical release from vesicles in the GUV model, release from the smaller plasma membrane vesicles result in faster release kinetics. The small-scale system and the fast kinetics make the visual observation of the membrane during release difficult. However, a large fraction of the events can be visually inspected. These observations lead to the finding that release in this model occurs through two distinct mechanisms. One mode, that we term full distension occurs when the nanotube connecting the daughter vesicle to the artificial cell membrane is completely extinguished, as the vesicle grows larger under pressure (Figure 2a). This results in the complete distension of the nanotube until it forms a frustum shaped connection between the pipette and the cell membrane. The frustum then reforms into a nanotube, leading to the expulsion of the entire vesicle content. The nanotube is again filled from the pipette tip end and fuses repeatedly in this manner. The second mode of release observed is one that to our knowledge has not been observed in previous model systems of exocytosis. In these events, the spherical part of the vesicle never reaches the cell membrane, but instead releases some of its content in a burst before it can accomplish full distension. We refer to this release mode as partial distension since the nanotube does not distend completely. Instead a wider tube is transiently formed, allowing partial release of the vesicle content. The result, as the pore again collapses into a nanotube, is a smaller vesicle still present at the tip of the micropipette and, importantly, only part of the content is released. This smaller vesicle is then refilled and the system fuses repeatedly this way (Figure 2b).

### Complete and partial distension modes display distinct release kinetics

**Figure 3:**
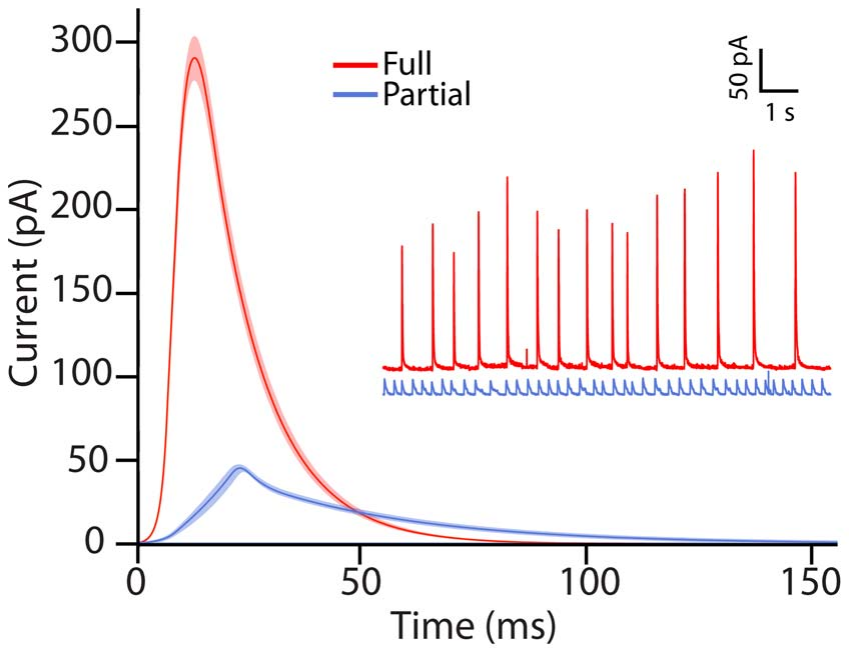
Representative traces and average peaks from the two modes of release. The average amperometric peaks for release though full (red) and partial (blue) distension plotted together with SEM. The inset shows representative amperometric traces for the two modes of release. Data were collected from 11 plasma membrane vesicles where recordings from 2 of these vesicles were defined as full release and 9 of them as partial release.

The release of dopamine from the artificial cell was monitored with carbon fiber amperometry. The release events were first analyzed based on our visual observations. This led to the assignment of two groups of events; one type that released through full distention of the nanotube and one that released through partial distension. The visual event assignments were then used to categorize the collected amperometic traces. In general it was rare to observe a full distension event in a trace containing partial events and likewise a partial distension event in a full release trace. Representative amperometric traces as well as the average amperometric peaks of the two release modes based on the visual characterization are shown in Figure 3. The amperometric peak, in addition to revealing the number of molecules released, provides detailed information on the kinetics of the release event with millisecond time resolution. The peaks were analyzed for peak current, half width (t_1/2_), identified as the width of the peak at half its maximum, rise time, which was defined as the time of rise from 25% to 75% of the peak height and fall time, equally defined as the time between 75% and 25% of the height of the peak. The average peaks for full versus partial opening display distinctly different kinetics and amounts of release with partial distension events releasing less neurotransmitter and having slower release kinetics when compared to the full distension events.

### Amperometric peak characteristics distinguish between two exocytosis modes

**Figure 4:**
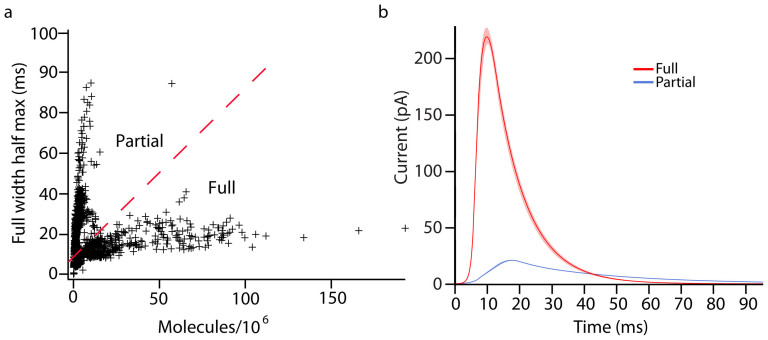
Distribution of release kinetics for all events and reassigned average peaks. (a). The full width at half max plotted against the number of molecules released for all detected peaks. The two distributions appear to result from partial and full distension modes of release. (b). Average peaks of the two modes with assignments based on amount and kinetics of release are plotted with SEM Data were collected from 11 plasma membrane vesicles where 5 traces were defined as full release and 6 as partial release.

Full peak width at half maximum, or half width, was plotted vs. the amount released to determine if the visual assignments of exocytosis modes correlated to two kinetically distinct modes of release. The half width of the release peak provides information about the duration of the event whereas the number of molecules released relates to the size of the vesicle. These data are plotted in Figure 4a showing two distinct populations; one with large half widths and small released amounts and one with small half widths and large amounts released. It appears that these two peak populations represent partial and full distension, respectively. In support of this hypothesis, we examined the initial assignments on an individual basis and found that they are generally accurate. However, these data were used to reassign some traces to the other mode. It is clearly difficult to define the event mode visually with 100% accuracy given the small sizes of the vesicles. The reassigned average peaks are displayed in Figure 4B showing an even greater distinction between the partial and full release modes.

**Figure 5:**
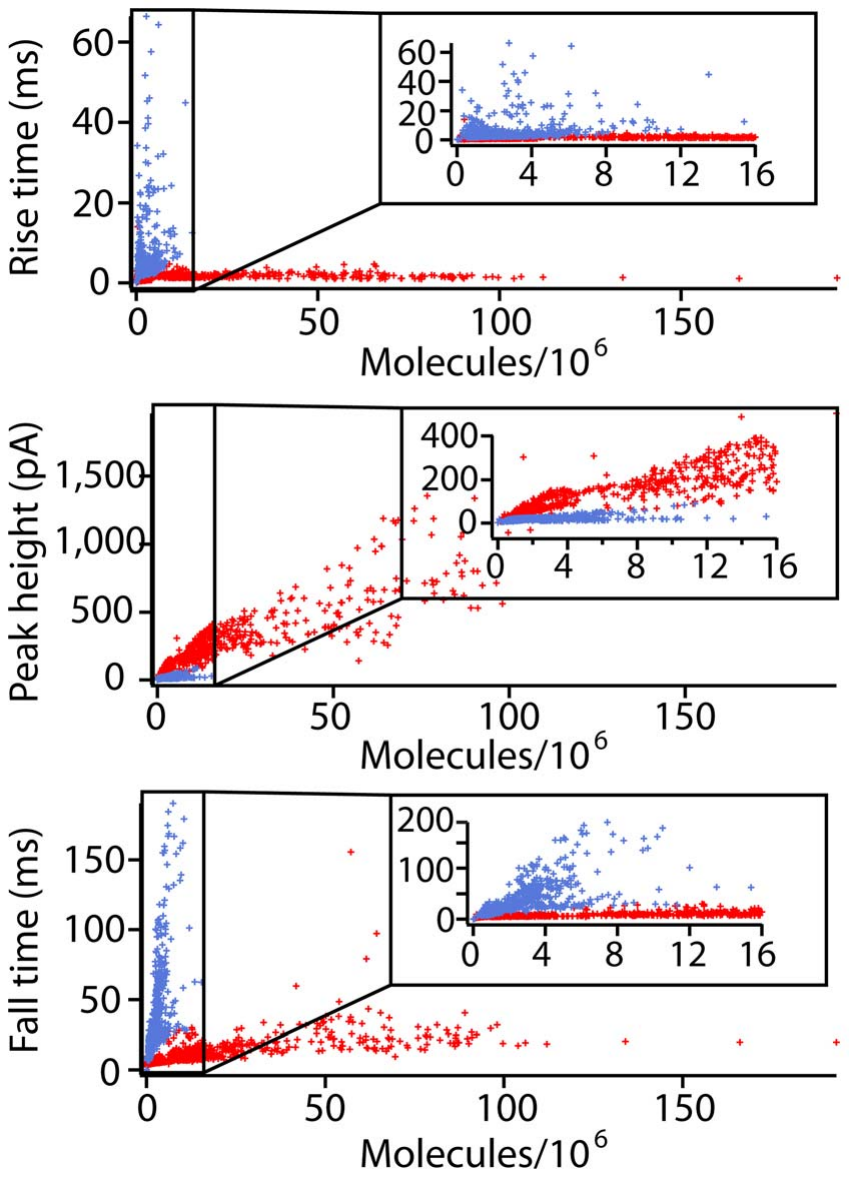
Vesicle opening dynamic parameters vs. charge for all events. The full distension events are labeled red while the partial distension events are blue. The inset of each graph is a blow up of the events with low released amounts.

Figure 5 shows data for three different spike parameters vs. the number of molecules detected at the electrode. The amount released is used here as an approximation of vesicle size as it is observed that for larger vesicles a larger amount is released, even in the case of partial distension events. First, rise time is compared to released amount; this parameter provides information about the opening stage of the pore. The data show no obvious trend; however, there is more variation in the partial release population. The next parameter compared is the peak height. The peak height represents the maximum flux and should indicate the maximum diameter of the fusion pore. This plot shows that the full release events increase the flux of dopamine as larger amounts are released. In contrast, the flux during partial release events is fairly constant. Finally, the fall time, which is taken as an indication of diffusion from the distended vesicle in the full fusion model and as pore closure in the partial release model has been examined. Here a strong relationship is observed between the amount released and the fall time in the partial case, whereas the relationship is less pronounced for vesicles undergoing full distention.

### Investigating membrane tension as a switching mechanism between the two modes of release

In previous work using artificial cells constructed from GUV membrane only the full distension mode of release has been observed. One aspect that separates the cell model system used in this work in comparison to the GUV system is a larger influence of membrane tension in the cell plasma membrane during the artificial exocytosis. The bleb is comprised of the membrane from a single cell and therefore has a finite surface area. Consequentially, manipulation of the bleb system causes changes in tension, which can influence the release mode.

**Figure 6:**
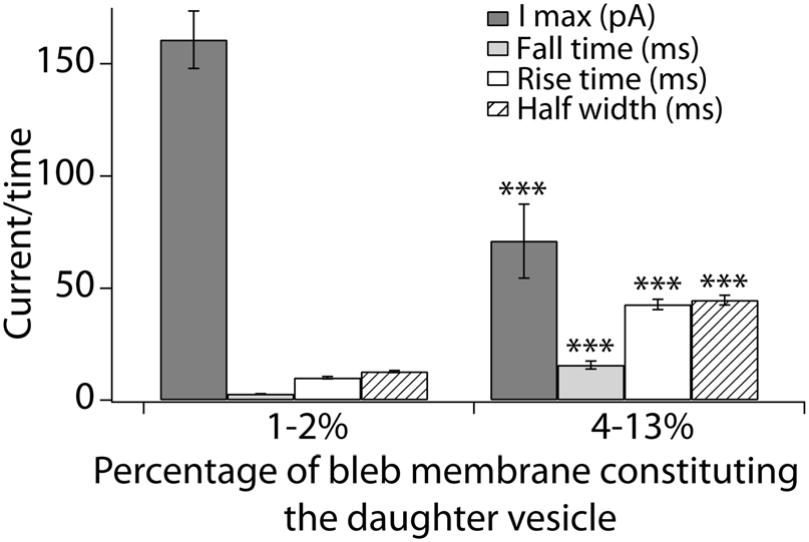
Switching between full and partial release in the same bleb. Peak characteristics of release events from two sizes of daughter vesicles formed inside the same bleb. The small vesicle displays a larger peak current and faster release kinetics compared to the larger daughter vesicle. The presented averages are from 4 separate blebs where both a small and a large vesicle were studied per bleb. *** p < 0.001 using t-test assuming equal variances.

In an attempt to investigate a possible regulating function of membrane tension in determining the release mode we studied release from two different sizes of daughter vesicles formed from the same bleb with the hypothesis that the differences in membrane tension between the two systems would result in different release modes. The smaller daughter vesicles comprised 1–2% of the entire bleb membrane area while the large daughter vesicles constituted 4–13% of the membrane area. The average amperometric peak characteristics are shown in Figure 6 where the peaks recorded from the smaller daughter vesicle are faster with higher amplitudes than the peaks recorded from the larger vesicle.

## Discussion

Current models of cellular exocytosis predominately investigate and assume the full distension type of release. The kinetics of this mode of release depends on a single opening event. Transient fusion with partial release of the vesicle content is known to frequently occur in secretory cells and neurons, and recent findings suggest that an extended form of kiss-and-run where the initial fusion pore expands only transiently, releasing a major part of the vesicle content, might be a major form of release in secretory cells^8^,^9^,^10^. In this mode of release, the kinetics are determined by two processes; fusion pore opening and closing. The observation of a second mode of release in our model is exciting as it shows the simplicity of this event taking place in the biological system and it can be used to study the parallel process of extended kiss-and-run release. Our model has the ability to mimic the expansion and subsequent restoration of the initial fusion pore, and the resultant partial release of the vesicle content.

Several characteristics of the partial release events that we have measured here can be used to draw conclusions about the hypothesized mechanism of extended kiss-and-run exocytosis. The most exciting finding is that the peak height of the partial release events is fairly uniform. The peak height represents the maximum flux of neurotransmitter through the pore, and it follows that the partial release events have a uniform pore size. This finding suggests that some independent factor determines the pore size, and it has been suggested that lipid composition might play a role here^12^,^20^. Furthermore, the kinetics of the events seems to be coupled to the size of the vesicle, specifically the fall time of the peak. The larger vesicles take a longer time to close than the smaller vesicles. Thus larger vesicles will release more cargo by extending the time of release in the partial distension mode while the full release mechanism increases flux as indicated by the increase in peak current. Finally we only see a weak connection between the rise time and released amount indicating again that some independent factor might determine the rate of pore expansion.

Interestingly, the artificial plasma membrane vesicle exocytosis experiment can be used to determine the factors that dictate the mode of release. Since the partial distension mode is not normally observed in artificial models of exocytosis, we were interested in investigating what separates the cell model system used here from previously used ones. Based on the limited amount of membrane material in our model, we hypothezised that membrane tension is a regulating factor. One aspect that affects the tension in the plasma membrane model is the size of the bleb. Since the daughter vesicles formed all range in size between 1 and 3 μm in diameter, a plasma membrane vesicle with a larger membrane surface area will result in a lower increase in tension during the vesicle release process. When studying the sizes of the mother vesicles that result in full and partial distension respectively, we find that the blebs releasing through the full distension mode are generally larger (p = 0.059) than those that release through partial distension, with an average diameter of the vesicles that release via full distension of 10.5 ± 0.7 μm compared to 9.3 ± 0.2 μm for vesicles that release through partial distension.

We speculate that there is a threshold in the mother vesicle size below which the tension differential during daughter vesicle growth is too large for full distention. Below this threshold in vesicle size, there is a limited amount of membrane to provide new material to the expanding daughter vesicle, which upon vesicle inflation increases the magnitude of the membrane stretching component significantly. Thus, in this situation partial distention is observed.

It appears that membrane tension is the determinant of release mode but the system is of course, also subject to other important forces such as hydrostatic pressure and membrane bending. The release mode will reflect a balance between all of the relevant forces. As noted, membrane bending energy makes the nanotube a stable energy minimum. Clearly, this minimum is realized only when the other forces are small. The hydrostatic pressure difference between the inside of the daughter vesicle and the outside of the bleb, together with the tension gradient, will force the nanotube to open in both modes, but with constant hydrostatic pressure and gradient (conditions here) the membrane tension would determine the differential mode.

**Figure 7:**
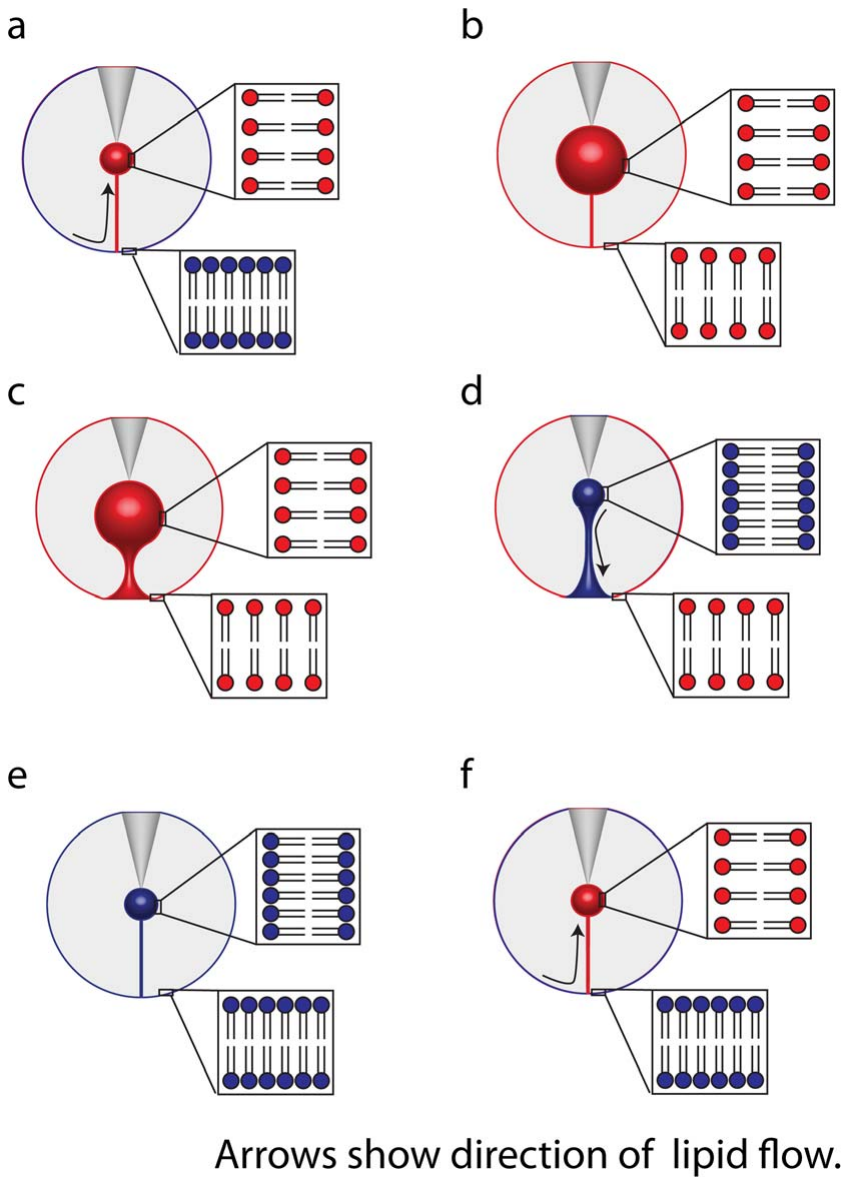
Proposed model for the tension dependence in partial release mode. The blue lipids symbolize a membrane at low tension while the red lipid bilayers symbolize a membrane under tension stress. (a). The inflation of the daughter vesicle creates tension in the vesicle membrane, inducing lipid flow from the mother to the daughter vesicle. (b). At some critical size the tension between the daughter and mother vesicles equalize, stopping flow. (c). The daughter vesicle inflation continues, which requires the mother vesicle to reduce surface area to accommodate the growing daughter vesicle (d). The pore formed reduces the pressure in the daughter vesicle stopping it from growing which causes a reverse in lipid flow. (e). The surface area is minimized as the toroid collapses to a lipid nanotube (f). The closing of the pore initiates the daughter vesicle inflation and the lipid flow starts again.

The concept of membrane tension as a driver for partial distention is demonstrated with a model in Figure 7. The inflation of the daughter vesicle induces Marangoni flow of lipid toward the pipette (Figure 7a)^21^. This accommodates the expanded surface and minimizes the increase in membrane tension caused during inflation. If there is no corresponding increase in lipid material, daughter vesicle expansion will be accompanied by increased tension over the entire system (Figure 7b). Plasma membranes can be expanded by approximately 2–3% in surface area without rupturing^22^,^23^. The daughter vesicles created here (1 to 3 μm diameter) contain approximately 4% of the total system membrane surface, in good agreement with this. When this tension is exceeded, the daughter vesicle cannot grow further and, hence, the nanotube expands into a toroid (Figure 7c). Content from the daughter vesicle is then released in a short burst. This shrinks the daughter vesicle diameter relieving membrane tension (Figure 7d). As the membrane tension equalizes over the system, the tube shrinks again to a nanotube (Figure 7e) corresponding to a state of minimum energy^24^. Once the tube is re-established the artificial exocytosis process starts over again (Figure 7f).

To test this model, we performed a separate experiment where the same bleb was used to study release from a small daughter vesicle and a large one. The formation of the small vesicle used between 1 and 2% of the total bleb membrane while the large vesicle comprised between 4 and 13% of the entire bleb membrane, placing the two structures on opposite sides of the hypothesized 2–3% limit. The amperometric results show that the release events from the smaller vesicle display the characteristics of full distension events with large peak currents and fast release dynamics while release from the larger vesicle is slower with lower peak heights indicating partial release. Furthermore, the amount released from the large set of vesicles was 1.6 times larger than the amount released from the smaller vesicles. This supports the hypothesis of large vesicle formation resulting in partial release and small vesicles releasing their full content as the difference in volume between the vesicle groups should theoretically result in a 12 time difference in released amount if they occurred through the same mode. These results are consistent with our proposed model suggesting that membrane tension dictates the mode of release. The data suggest that when the tension differential is high between the releasing vesicle and the cell, the release event is governed by an open-and-closed mechanism. Membrane tension driving the opening of the fusion pore and release is not surprising^25^,^26^. However, the data and model shown here suggest a mechanism for the membrane pore closing again. The artificial cell experimental paradigm has the unique ability to interrogate and isolate the independent factors controlling the two release modes and furthermore suggests membrane tension as a key player in regulation of the fusion pore dynamics during exocytosis.

In conclusion, we present an artificial model for exocytosis constructed from plasma membrane material obtained from a living cell. With video microscopy and simultaneous amperometric detection of vesicular dopamine release from this cell, two different modes of artificial exocytosis are observed. One that approximates the classic full distention of the vesicle membrane, and one that is similar to kiss-and-run exocytosis, but extended in time and opening. In our artificial cell, the membrane must expand to accommodate the filling of the daughter vesicle. The increase in tension caused by this expansion may cause a premature pore opening, which is simulated as a partial release event. The mode of release that occurs in this model appears to be dictated by the membrane tension. The partial distension mode is further shown to regulate the amount released by altering the time that it takes to close the pore. This model is attractive as the membrane retains much of its initial composition, making it a more lifelike system as compared to the lipid extract commonly used in models such as this.

## Methods

### Cell culture and formation of plasma membrane blebs

PC12 cells were obtained from the American Type Tissue Culture Collection and maintained as previously described^27^. Briefly, cells were cultured on collagen type IV coated flasks (BD BioCoat™) and sub cultured when confluence was reached, approximately every 7 days. Cells were grown in RPMI-1640 medium supplemented with 10% donor horse serum and 5% fetal bovine serum. The medium was changed every 2–3 days. For experiments, cells were plated on glass bottom dishes (Willco Wells) coated with collagen type IV (Sigma Aldrich). Formation of plasma membrane vesicles was induced by a 30 min incubation of the cells in a blebbing solution (25 mM formaldehyde, 2 mM dithiothreitol (DTT), 10 mM HEPES, 2 mM CaCl_2_ and 150 mM NaCl, pH 7.4)^28^. After incubation, the cells were washed three times in HEPES buffer (10 mM HEPES, 140 mM NaCl, 1 mM CaCl_2_, 5 mM KCl and 1 mM MgCl_2_, 10 mM D-glucose, pH 7.4) and finally 2 mL of HEPES buffer was added for experiments.

### Artificial release in blebs

Experiments were performed as previously described^16^. Briefly, a micropipette (o.d. 1 mm, i.d. 0.78 mm, with filament; Harvard Apparatus, Holliston, MA) was pulled on a commercial filament puller (P-1000; Sutter Instrument Co., Novato, CA) using a Type C program yielding pipettes 0.8–0.5 μm and 40–80 M ohm. The resistance was periodically measured using an axoscope 200 B and was found to consistency fall within this range. The pipette was electro-inserted into a plasma membrane vesicle and further electroporated through the second membrane of the vesicle so that the pipette tip was sticking out through the membrane on the opposite side of the insertion. The pipette was then retracted back through the second membrane, bringing with it a tube of lipid. This nanometric tube connects the inside of the pipette with the outside of the bleb. By applying a constant backpressure of 40–50 hPa through the pipette, using a FemtoJet microinjector (Eppendorf/Brinkmann Instruments, Hauppauge, NY), the tube was inflated to form a vesicle at the tip of the pipette. Prior to experiments, the pipette was backfilled with 50 mM dopamine dissolved in a buffer solution (5 mM Trizma base, 15 mM K_3_PO_4_, 30 mM KH_2_PO_4_, 10 mM K_2_HPO_4_, 0.5 mM EDTA and 1 mM MgSO_4_ at pH 7.4).

### Carbon fiber amperometry

Release from plasma membrane vesicles was studied using amperometry at a carbon fiber microelectrode placed at the exit of the nanotube. The microelectrodes were constructed by aspirating a 5-μm carbon fiber into a glass capillary (o.d. 1.2 mm i.d. 0.69 mm, no filament; Sutter Instrument Co., Novato, CA). The capillary was pulled with a commercial micropipette puller (PE-21, Narishige, Japan) producing two fiber-containing pipettes. The protruding carbon fiber was cut with a scalpel close to the glass tip and dipped into freshly made epoxy (EpoTek 301, Billerica, MA) for 1 min, creating a seal between the carbon fiber and the glass. The epoxy was allowed to cure and the electrodes were then polished at a 45° angle on a commercial micropipette beveler (Narishige, Japan) and backfilled with 3 M KCl^29^. Prior to experiments, electrodes were tested in 100 mM dopamine solution and only electrodes with stable I–E curves were used. For amperometric measurements, the electrode was held at 700 mV vs. a silver/silver chloride reference electrode (World Precision Instruments, Inc., Sarasota, FL) using a commercial instrument (Axopatch 200B; Axon Instruments, Foster City, CA). The signal was digitized at 5 kHz and filtered with an internal lowpass Bessel filter at 2 kHz. The signal was displayed in real time (AxoScope 8.1; Axon Instruments) and stored digitally.

### Microscopy

Experiments were monitored using an Olympus IX-71 or IX-81 microscope (Olympus, Melville, NY) with a 40× oil objective (Olympus, UApo/340 40× oil iris, NA 1.35). Differential interference contrast (DIC) was utilized for a pseudo-three dimensional appearance and contrast enhancement of the bleb. An Olympus SC20 digital color camera interfaced to a personal computer with the Cell-A software (Olympus, Hamburg, Germany) was used for visual recording of the experiments.

### Data analysis and statistics

The amperometric data were analyzed in Igor Pro 6 (Version 6.2.2.0; WaveMetrics, Lake Oswego, OR) using an Igor Procedure File designed for analysis of quantal release by the group of David Sulzer^30^. Vesicle size measurements were performed with the NIH developed ImageJ software where the diameter of each vesicle was measured 5 times and an average was calculated. Data were tested for significant differences using two-tailed t-test assuming equal variances.

## Author Contributions

L.J.M. and N.N. performed all experiments. M.E.K., L.J.M. and J.D. analysed and interpreted the data; L.J.M., M.E.K. and J.D. prepared all the figures. L.J.M., N.N., M.E.K. and A.S.C. designed the experiments for the development of the artificial cell model using blebbing cell plasma membrane. L.J.M. and M.E.K. wrote the main part of the manuscript together with A.G.E. and A.S.C. All authors read and approved the manuscript.

## References

[b1] Alvarez de Toledo G., Fernandez-Chacon R. & Fernandez J. M. Release of secretory products during transient vesicle fusion. Nature. 363, 554–558 (1993). 8505984 10.1038/363554a0

[b2] Wightman R. M. & Haynes C. L. Synaptic vesicles really do kiss and run. Nat Neurosci. 7, 321–322 (2004). 15048116 10.1038/nn0404-321

[b3] Fesce R., Grohovaz F., Valtorta F. & Meldolesi J. Neurotransmitter release: fusion or ‘kiss-and-run'? Trends Cell Biol. 4, 1–4 (1994). 14731821 10.1016/0962-8924(94)90025-6

[b4] Stevens C. F. & Williams J. H. “Kiss and run” exocytosis at hippocampal synapses. Proc Natl Acad Sci USA. 97, 12828–12833 (2000). 11050187 10.1073/pnas.230438697 PMC18849

[b5] Elhamdani A., Azizi F. & Artalejo C. R. Double patch clamp reveals that transient fusion (kiss-and-run) is a major mechanism of secretion in calf adrenal chromaffin cells: high calcium shifts the mechanism from kiss-and-run to complete fusion. J Neurosci. 26, 3030–3036 (2006). 16540581 10.1523/JNEUROSCI.5275-05.2006 PMC6673983

[b6] Staal R. G., Mosharov E. V. & Sulzer D. Dopamine neurons release transmitter via a flickering fusion pore. Nat Neurosci. 7, 341–346 (2004). 14990933 10.1038/nn1205

[b7] Elhamdani A., Palfrey H. C. & Artalejo C. R. Quantal size is dependent on stimulation frequency and calcium entry in calf chromaffin cells. Neuron. 31, 819–830 (2001). 11567619 10.1016/s0896-6273(01)00418-4

[b8] Mellander L. J., Trouillon R., Svensson M. I. & Ewing A. G. Amperometric post spike feet reveal most exocytosis is via extended kiss-and-run fusion. Sci Rep. 2, 907 (2012). 23205269 10.1038/srep00907 PMC3510463

[b9] Omiatek D. M., Dong Y., Heien M. L. & Ewing A. G. Only a Fraction of Quantal Content is Released During Exocytosis as Revealed by Electrochemical Cytometry of Secretory Vesicles. ACS Chem Neurosci. 1, 234–245 (2010). 20368748 10.1021/cn900040e PMC2847285

[b10] Omiatek D. M. *et al.* The real catecholamine content of secretory vesicles in the CNS revealed by electrochemical cytometry. Sci Rep. 3, 1447 (2013). 23486177 10.1038/srep01447 PMC3596796

[b11] Omiatek D. M., Santillo M. F., Heien M. L. & Ewing A. G. Hybrid capillary-microfluidic device for the separation, lysis, and electrochemical detection of vesicles. Anal Chem. 81, 2294–2302 (2009). 19228035 10.1021/ac802466g PMC2656409

[b12] Amatore C. *et al.* Regulation of exocytosis in chromaffin cells by trans-insertion of lysophosphatidylcholine and arachidonic acid into the outer leaflet of the cell membrane. Chembiochem. 7, 1998–2003 (2006). 17086558 10.1002/cbic.200600194

[b13] Haynes C. L., Siff L. N. & Wightman R. M. Temperature-dependent differences between readily releasable and reserve pool vesicles in chromaffin cells. Biochim Biophys Acta. 1773, 728–735 (2007). 17467077 10.1016/j.bbamcr.2007.03.013 PMC2025685

[b14] Pihel K., Travis E. R., Borges R. & Wightman R. M. Exocytotic release from individual granules exhibits similar properties at mast and chromaffin cells. Biophys J. 71, 1633–1640 (1996). 8874038 10.1016/S0006-3495(96)79368-2 PMC1233631

[b15] Schroeder T. J. *et al.* Temporally resolved, independent stages of individual exocytotic secretion events. Biophys J. 70, 1061–1068 (1996). 8789125 10.1016/S0006-3495(96)79652-2 PMC1225008

[b16] Cans A. S. *et al.* Artificial cells: unique insights into exocytosis using liposomes and lipid nanotubes. Proc Natl Acad Sci USA. 100, 400–404 (2003). 12514323 10.1073/pnas.232702599 PMC141006

[b17] Wittenberg N. J., Zheng L., Winograd N. & Ewing A. G. Short-chain alcohols promote accelerated membrane distention in a dynamic liposome model of exocytosis. Langmuir. 24, 2637–2642 (2008). 18278956 10.1021/la703171u PMC2553711

[b18] Scott R. E. Plasma membrane vesiculation: a new technique for isolation of plasma membranes. Science. 194, 743–745 (1976). 982044 10.1126/science.982044

[b19] Tank D. W., Wu E. S. & Webb W. W. Enhanced molecular diffusibility in muscle membrane blebs: release of lateral constraints. J Cell Biol. 92, 207–212 (1982). 7199052 10.1083/jcb.92.1.207 PMC2112013

[b20] Amatore C. *et al.* Relationship between amperometric pre-spike feet and secretion granule composition in chromaffin cells: an overview. Biophys Chem. 129, 181–189 (2007). 17587484 10.1016/j.bpc.2007.05.018

[b21] Staykova M., Lipowsky R. & Dimova R. Membrane flow patterns in multicomponent giant vesicles induced by alternating electric fields. Soft Matter. 4, 2168–2171 (2008). 22096459 10.1039/b811876k PMC2898647

[b22] Needham D. & Hochmuth R. M. Electro-mechanical permeabilization of lipid vesicles. Role of membrane tension and compressibility. Biophys J. 55, 1001–1009 (1989). 2720075 10.1016/S0006-3495(89)82898-X PMC1330536

[b23] Vlahakis N. E. & Hubmayr R. D. Invited review: plasma membrane stress failure in alveolar epithelial cells. J Appl Physiol. 89, 2490–2496 (2000). 11090606 10.1152/jappl.2000.89.6.2490

[b24] Iglic A., Kralj-Iglic V. & Majhenc J. Cylindrical shapes of closed lipid bilayer structures correspond to an extreme area difference between the two monolayers of the bilayer. J Biomech. 32, 1343–1347 (1999). 10569713 10.1016/s0021-9290(99)00136-0

[b25] Amatore C. *et al.* Correlation between vesicle quantal size and fusion pore release in chromaffin cell exocytosis. Biophys J. 88, 4411–4420 (2005). 15792983 10.1529/biophysj.104.053736 PMC1305668

[b26] Amatore C. *et al.* Dynamics of full fusion during vesicular exocytotic events: release of adrenaline by chromaffin cells. Chemphyschem. 4, 147–154 (2003). 12619413 10.1002/cphc.200390024

[b27] Kozminski K. D., Gutman D. A., Davila V., Sulzer D. & Ewing A. G. Voltammetric and pharmacological characterization of dopamine release from single exocytotic events at rat pheochromocytoma (PC12) cells. Anal Chem. 70, 3123–3130 (1998). 11013717 10.1021/ac980129f

[b28] Stepanyants N., Jeffries G. D., Orwar O. & Jesorka A. Radial sizing of lipid nanotubes using membrane displacement analysis. Nano Lett. 12, 1372–1378 (2012). 22313341 10.1021/nl203983e PMC3303199

[b29] Chen T. K., Luo G. & Ewing A. G. Amperometric monitoring of stimulated catecholamine release from rat pheochromocytoma (PC12) cells at the zeptomole level. Anal Chem. 66, 3031–3035 (1994). 7978300 10.1021/ac00091a007

[b30] Mosharov E. V. & Sulzer D. Analysis of exocytotic events recorded by amperometry. Nat Methods. 2, 651–658 (2005). 16118635 10.1038/nmeth782

